# Adolescent conditioning affects rate of adult fear, safety and reward learning during discriminative conditioning

**DOI:** 10.1038/s41598-018-35678-9

**Published:** 2018-11-23

**Authors:** Iris Müller, Alyson L. Brinkman, Elizabeth M. Sowinski, Susan Sangha

**Affiliations:** 1 Department of Psychological Sciences, Purdue University, West Lafayette, IN 47907-2081 USA; 2 Purdue Institute for Integrative Neuroscience, Purdue University, West Lafayette, IN 47907-2081 USA

## Abstract

Fear and reward memories formed in adulthood are influenced by prior experiences. Experiences that occur during sensitive periods, such as adolescence, can have an especially high impact on later learning. Fear and reward memories form when aversive or appetitive events co-occur with initially neutral stimuli, that then gain negative or positive emotional load. Fear and reward seeking behaviours are influenced by safety cues, signalling the non-occurrence of a threat. It is unclear how adolescent fear or reward pre-conditioning influences later dynamics of these conditioned emotions, and conditioned safety. In this study, we presented male rats with adolescent fear or reward pre-conditioning, followed by discriminative conditioning in adulthood. In this discriminative task, rats are simultaneously conditioned to reward, fear and safety cues. We show that adolescent reward pre-conditioning did not affect the rate of adult reward conditioning, but instead accelerated adult safety conditioning. Adolescent fear pre-conditioning accelerated adult fear and reward seeking behaviours but delayed adult safety expression. Together, our results suggest that the dynamics of safety conditioning can be influenced by adolescent priming of different valences. Taking adolescent experiences into consideration can have implications on how we approach therapy options for later learned fear disorders where safety learning is compromised.

## Introduction

Two main environmental determinants of an individual´s behaviour are cues signalling threat and reward, thus inducing fear and reward seeking, respectively. Dysregulations in processing these stimuli can lead to psychiatric conditions, like posttraumatic stress disorder (PTSD) and addiction. Not only does the comorbidity between these disorders^1,2^ suggest substantial interaction of reward and fear processing systems, rodent models have also established a tight cross talk on a behavioural^3^, molecular (rev. in^4^) and anatomical/circuit level^5–12^. One of the best researched structures mediating the association of stimuli with different emotional loads is the basolateral amygdala, harbouring neurons responsive to reward and threat predictive cues^5,7–10^. But other brain regions, like the ventral tegmental area^6^, paraventricular thalamus^12^ and the nucleus accumbens^11^ have also been identified as common substrates for reward and aversion processing.

A significant determinant of PTSD and addiction are prior experiences made during sensitive developmental periods, like adolescence^13,14^. Similar to humans, experiences in rodent adolescence (roughly postnatal day P21 (after weaning) to P42 (onset of sexual reproduction)^15^), whether they are positive or negative, shape later fear and reward related behaviours^16–20^. Adolescent exposure to severe stress leads to behavioural abnormalities^16,17,20^, reminiscent of the human phenotype of PTSD, but positive experiences can protect against a pathological development^18^. Moreover, previous experiences also influence the interplay of these systems. For example, Bolton *et al*.^21^ showed that pre-weaning stress in rats leads to the recruitment of a fear-mediating neuronal population in the amygdala during rewarding social play behaviour in adolescence^21^.

Interestingly, but less well understood, both behaviours are influenced by safety cues, which signal the absence of a threat. Safety conditioning finds application in psychotherapy of PTSD, together with the repeated unreinforced exposure of threat associated stimuli, i.e. extinction^22,23^. While the fear suppressing effect of safety cues has been replicated in different rodent paradigms^7,24,25^, studies investigating the influence of safety cues on reward seeking have yielded controversial results, showing either no effect^26^ or a positive influence, as shown by an increase of lever presses for food rewards^27^. In the basal amygdala, a neuronal subpopulation encoding both reward and safety cues has been identified^7^, and reward circuits are engaged in active avoidance learning, when an animal shuttles to the safe compartment of a conditioning chamber^28^.

It is thus tempting to speculate that adolescent fear and reward conditioning influences subsequent learning of the same and opposite valences, and that both will impact later safety conditioning. To test this hypothesis we employed adolescent paired fear, unpaired fear or reward conditioning followed by discriminative conditioning (DC^7^) in adulthood. In this DC task, rats learn to distinguish between fear and reward cues, and to suppress their fear response when the fear cue is paired with a safety cue that signals the absence of shock. To assess the dynamics of and interactions between the different valences in the DC-task that are potentially influenced by previous experiences, adolescent rats were pre-conditioned to just one association of the DC-task. For example, the pre-conditioned reward group received the same reward cue-sucrose pairing in both adolescence and adulthood. We show that adolescent reward conditioning had no effect on adult reward learning, but accelerated safety learning. Adolescent fear conditioning accelerated adult fear and reward-seeking behaviours, as well as fear extinction, but delayed safety learning. Together, our results suggest that the dynamics of safety conditioning are influenced by pre-conditioning of different valences. Understanding the complex interaction of emotionally-charged cues within an individual’s history will advance our understanding of the heterogenic clinical picture in PTSD and addiction.

## Results

### Adolescent experiences did not alter baseline measures

**Figure 1:**
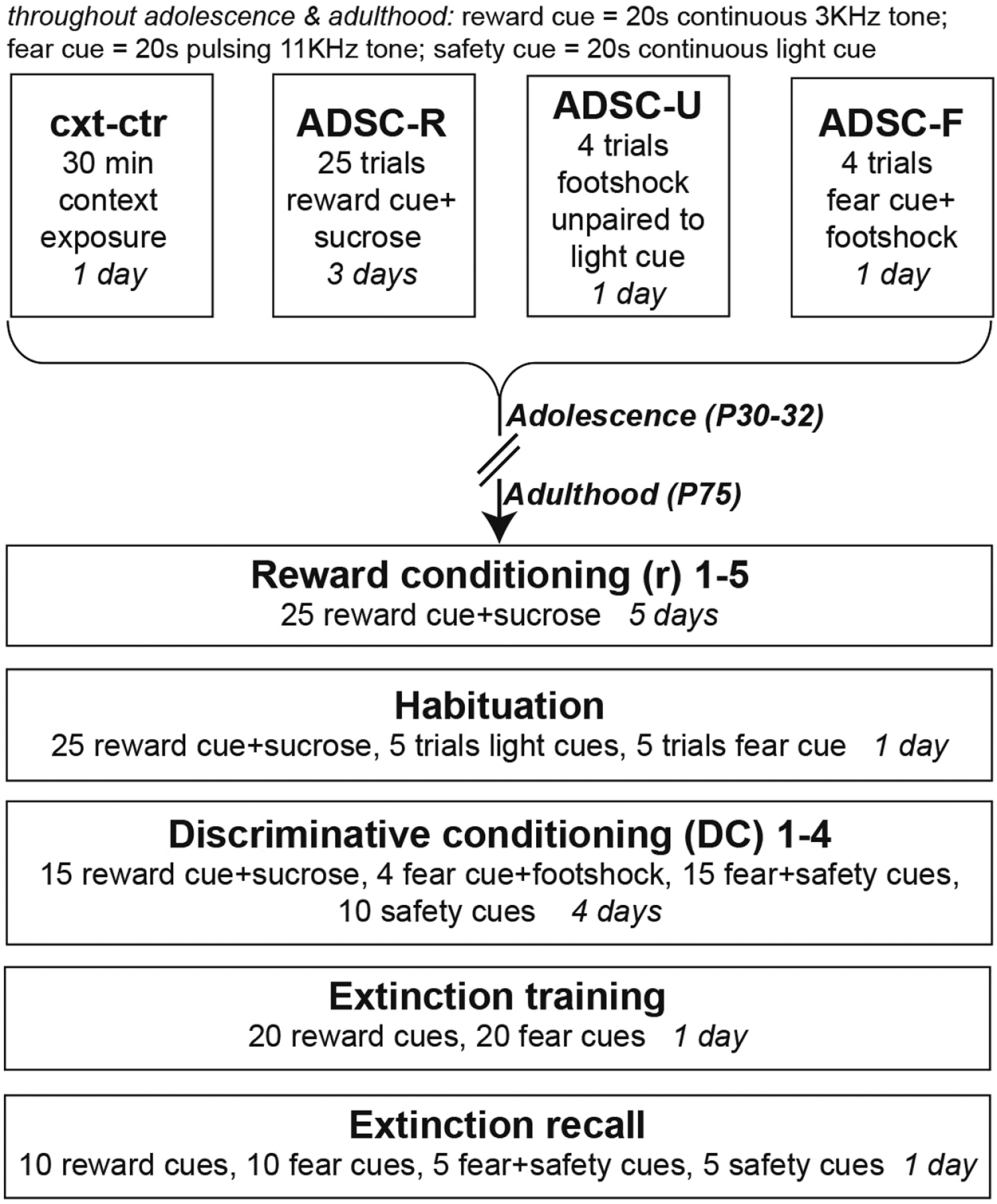
Paradigm outline. In adolescence (P30), rats were exposed to the future conditioning context alone, reward conditioning, to the safety cue unpaired to footshocks, or fear conditioning (cxt-ctr, ADSC-R, ADSC-U, ADSC-F). In adulthood (P75), rats received reward conditioning (r), followed by 1 habituation session to familiarize rats to the later fear and safety cues. Next, rats underwent 4 days of discriminative conditioning (DC) followed by extinction training and extinction recall.

During adolescence, rats were exposed to presentations of the reward cue paired with sucrose (ADSC-R), the fear cue paired with footshock (ADSC-F), footshocks unpaired to the safety cue (ADSC-U), or the context alone (ctr-cxt) (Fig. 1). Adolescent conditioning procedures did not affect body weight gain, indicating no unspecific stress effects of the adolescent experiences. All groups showed a uniform increase throughout development (two-way RM ANOVA: time: F(5, 300) = 5921, p < 0.0001; group: F(3, 60) = 0.345, p = 0.7929; interaction: F(15, 300) = 0.7233, p = 0.7605) and similar body weight gain during adult conditioning (time: F(15, 900) = 991.4, p < 0.0001; group: F(3, 60) = 0.5615, p = 0.6425; interaction: F(45, 900) = 0.6159, p = 0.9784). More importantly, the adolescent experience did not alter responding to the first adult presentation of the fear or safety cues in the habituation session (one-way ANOVA: fear cue: % time in the port: F(3, 60) = 0.5283, p = 0.6646; % time freezing: F(3, 60) = 0.5771, p = 0.6323; safety cue: % time in the port: F(3, 60) = 0.4834, p = 0.6951; % time freezing: F(3, 60) = 0.4337, p = 0.7296). A detailed analysis of freezing to the first adult presentation of individual reward, fear and safety cues can be found in Supplementary Fig. S1.

### Adolescent fear conditioning accelerated adult reward conditioning

**Figure 2:**
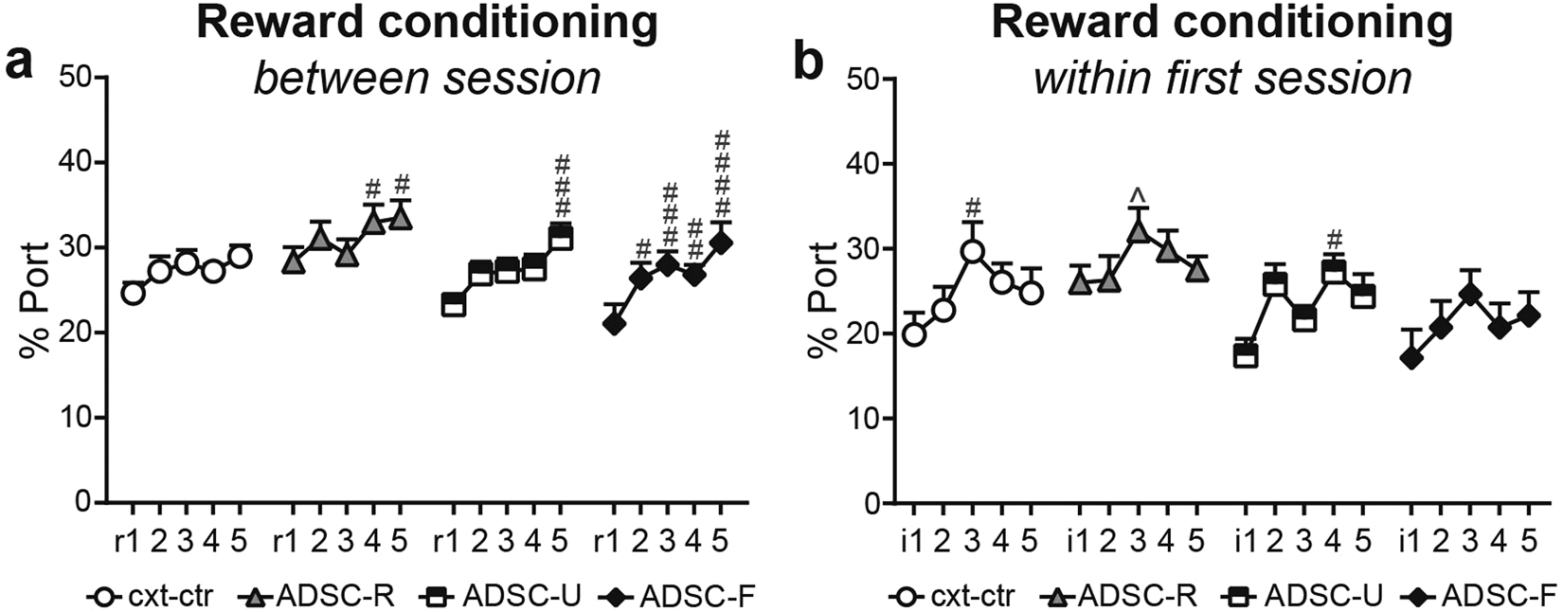
Adolescent fear conditioning accelerated adult reward conditioning. Averaged % time spent in the port during each reward cue was used to assess reward seeking behaviour. Over the 5 days of reward conditioning (r1-5) ADSC-F rats presented the steepest learning curve, despite insignificant baseline differences at r1 (**a**). This effect in the ADSC-F group was not already apparent within the first reward conditioning session (**b**). r1-5: reward session 1-5. i: interval, 5 consecutive reward trials were averaged to form an interval; ^####^p < 0.0001, ^###^p < 0.001, ^##^p < 0.01 and ^#^p < 0.05 vs r1 (a) or i1 (b), ^p < 0.05 vs ADSC-U of the same interval. Data are mean + SEM.

During five days of adult reward conditioning, all groups developed a significant increase in % time in the port (Fig. 2a; two-way RM ANOVA: session: F(4, 240) = 16.460, p < 0.0001; group: F(3, 60) = 2.332, p = 0.0832; interaction: F(12, 240) = 1.064, p = 0.3911). Tukey´s post hoc comparisons revealed the steepest learning curve for the ADSC-F rats (adolescent-fear group); unlike other groups, ADSC-F rats significantly increased reward seeking from r1 to r2, despite insignificant baseline differences at r1 among the groups (ADSC-F: r1 vs r2: p = 0.0145, r1 vs r3: p = 0.0005, r1 vs r4: p = 0.0065, r1 vs r5: p < 0.0001; cxt-ctr: r1 vs r5: p = 0.0751; ADSC-R: r1 vs r4: p = 0.0481, r1 vs r5: p = 0.0189; ADSC-U: r1 vs r5: p < 0.0001). In the ADSC-F group, this effect was not yet apparent within the first reward conditioning session. Two-way RM-ANOVA indicated a significant main effect for interval (Fig 2b; F(4, 240) = 5.714, p = 0.0002) and group (F(3, 60) = 3.323, p = 0.0256), but no interaction (F(12, 240) = 0.9861, p = 0.4627).The cxt-ctr group increased its time in the port from intervals (average of 5 trials) i1 to i3 (p = 0.0173) and the ADSC-U group from i1 to i4 (p = 0.0158). During i3 the ADSC-R group differed from ADSC-U rats (p = 0.0244).

### Adolescent conditioning did not affect reward seeking during adult discriminative conditioning or reward extinction

**Figure 3:**
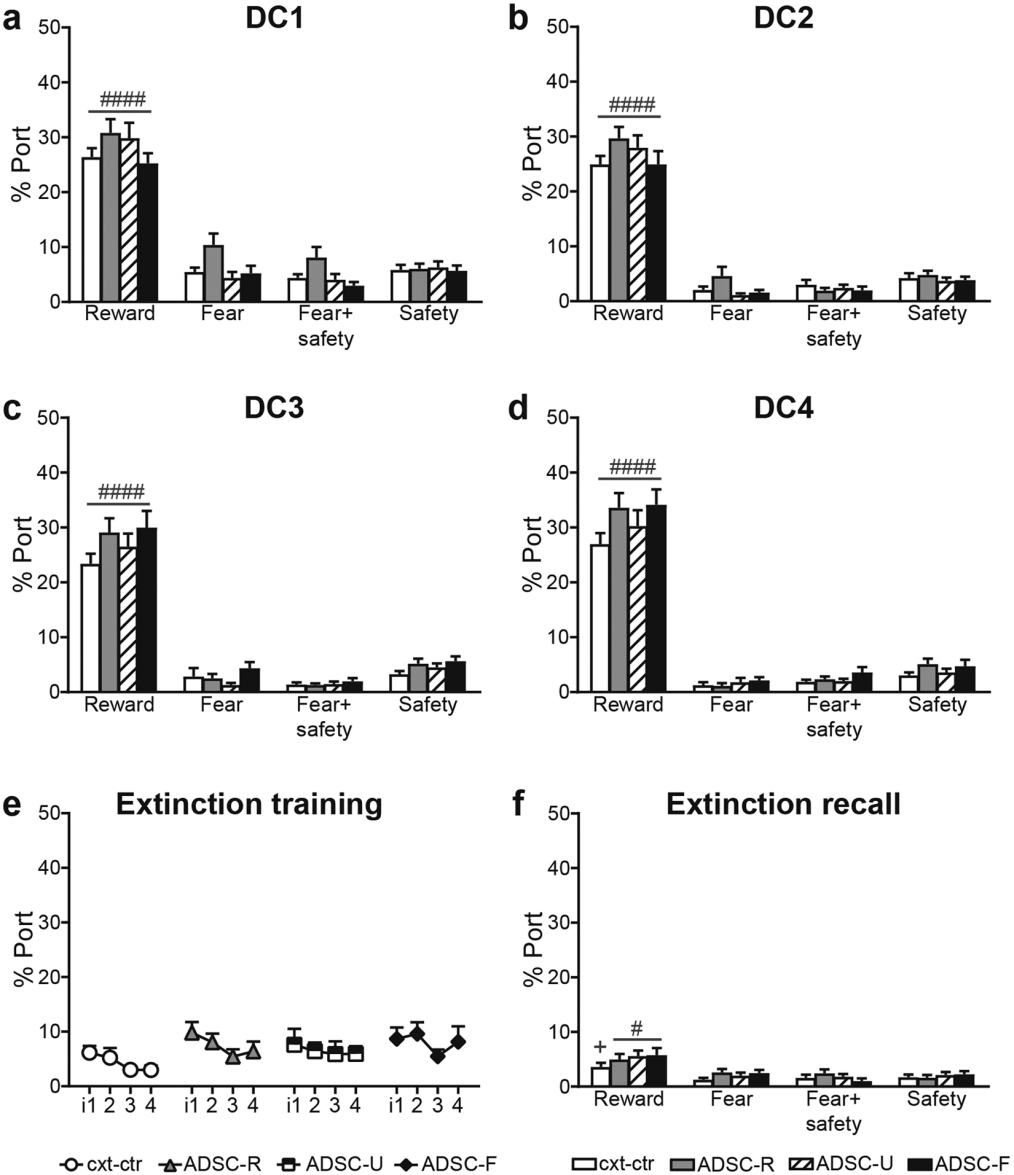
Adolescent conditioning does not affect reward seeking during adult discriminative conditioning (DC) or reward extinction. Throughout DC all rats spent more time in the port upon reward cue presentation than upon presentation of any other cue (**a**–**d**). During extinction, all groups significantly and uniformly reduced their time in the port (**e**), which persisted to the extinction recall (**f**). i: interval, 5 consecutive reward trials were averaged to form an interval; ^####^p < 0.0001 and ^#^p < 0.05: reward vs all other cues, ^+^p < 0.05 vs fear cue of the same group. Data are mean + SEM.

Throughout discriminative conditioning, all groups displayed significantly more time in the port upon reward cue presentation compared to any other cue (Fig. 3a–d; two-way RM ANOVAs: DC1: cue: F(3, 180) = 311.9, p < 0.0001; group: F(3, 60) = 2.715, p = 0.0526; interaction: F(9, 180) = 1.479, p = 0.1586; DC2: cue: F(3, 180) = 494, p < 0.0001; group: F(3, 60) = 1.284, p = 0.2880; interaction: F(9, 180) = 1.409, p = 0.1869; DC3: cue: F(3, 180) = 369.8, p < 0.0001; group: F(3, 60) = 1.775, p = 0.1616; interaction: F(9, 180) = 1.164, p = 0.3206; DC4: cue: F(3, 180) = 463.3, p < 0.0001; group: F(3, 60) = 2.099, p = 0.1099; interaction: F(9, 180) = 1.202, p = 0.2964; post hoc comparisons: reward cue vs all other cues: p < 0.0001 for all groups).

Extinction training induced a uniform and significant decrease in % time spent in the port in all groups (Fig. 3e; two-way RM ANOVA: interval: F(3, 180) = 3.633, p = 0.0140; group: F(3, 60) = 1.446, p = 0.2383; interaction: F(9, 180) = 0.3336, p = 0.9629). During extinction recall on the next day (Fig. 3f), all rats, with the exception of the cxt-ctr group, still spent more time in the port during the reward cue than during the other three cues (two-way RM ANOVA: cue: F(3, 180) = 23.68, p < 0.0001; group: F(3, 60) = 0.9596, p = 0.4178; interaction: F(9, 180) = 0.8552, p = 0.5665; post hoc comparisons: reward cue vs all other cues: p < 0.05 for ADSC-R, ADSC-U and ADSC-F; reward cue vs fear cue: p = 0.0498 for the cxt-ctr group). Importantly however, percent time in the port was significantly lower during reward cue presentation in the extinction recall (Fig. 3f) than at the beginning of extinction training (i1; Fig. 3e) for all groups, indicating successful extinction (two-way RM ANOVA with i1, and reward cue in the extinction recall as the repeated factor: extinction stage: F(1, 60) = 29.97, p < 0.0001; group: F(3, 60) = 0.9348, p = 0.4295; interaction: F(3, 60) = 0.2091, p = 0.8897).

### Adolescent conditioning affected fear expression and rate of safety expression during adult discriminative conditioning

**Figure 4:**
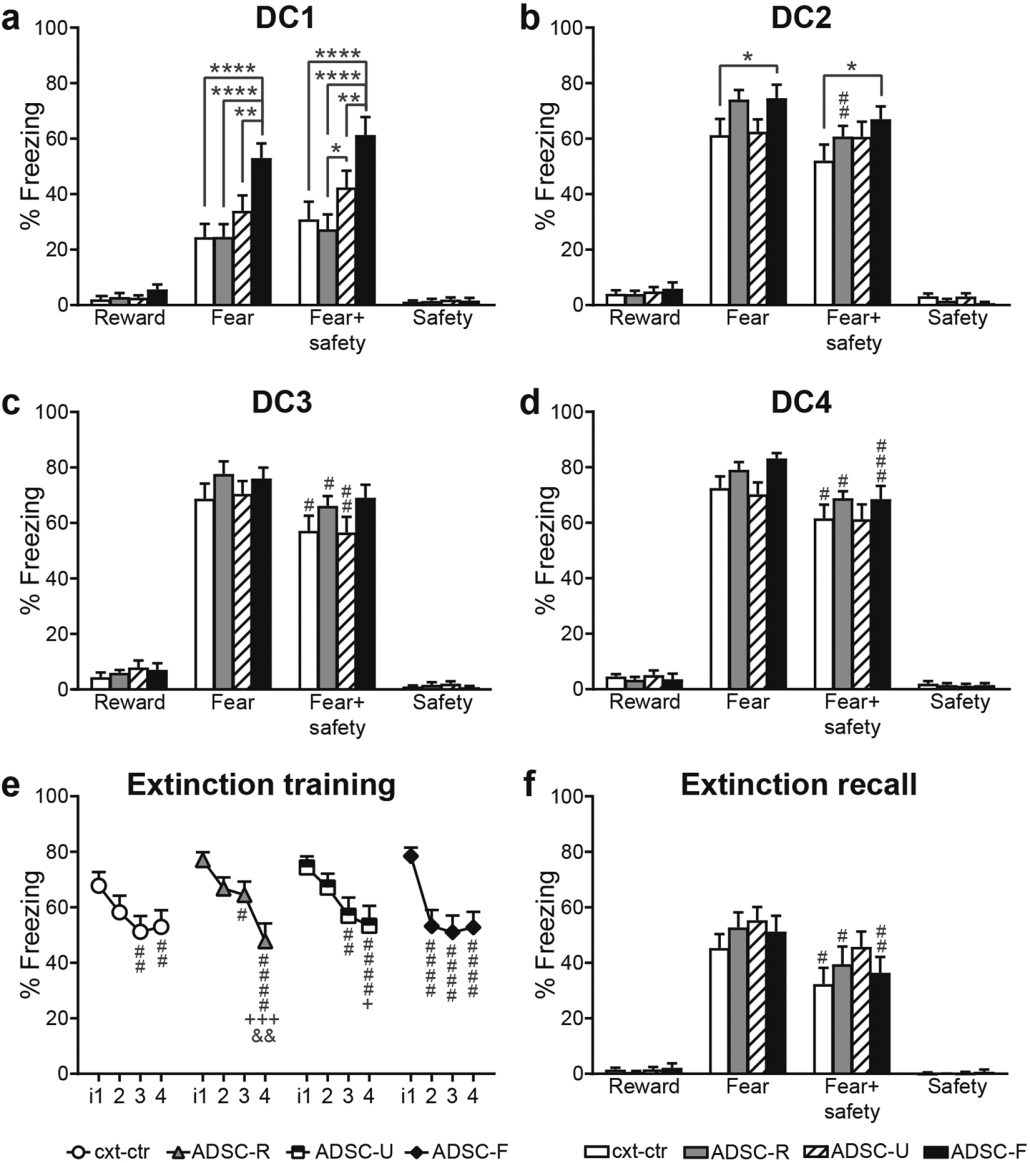
Adolescent conditioning affects fear expression and rate of safety learning during adult discriminative conditioning. Averaged % time freezing during each cue was used to assess fear related behaviour. The ADSC-F group showed increased freezing to the fear and the fear + safety cue in DC1 and 2 (**a**,**b**). Safety learning is defined as a significant suppression of freezing to the fear + safety cue, compared to the fear cue alone. ADSC-R rats showed significant safety learning as early as DC2 (**b**–**d**), whereas the ADSC-F group did not show significance until DC4 (**d**). The cxt-ctr group showed safety learning from DC3 onwards (**c**,**d**) and the ADSC-U group only in DC3 (**c**,**d**). During extinction, all groups reduced their fear response, but at different rates (**e**) and in the extinction recall fear suppression was maintained in all groups except the ADSC-U group (**f**). i: interval, 5 consecutive fear trials were averaged to form an interval; ****p < 0.0001, **p < 0.01, *p < 0.05 group differences within cue, ^####^p < 0.0001, ^###^p < 0.001, ^##^p < 0.01, ^#^p < 0.05 fear + safety cue vs fear within group (**a**–**d**,**f**) or vs i1 within group (**e**), ^+++^p < 0.001, ^+^p < 0.05 vs i2 within group (**e**) and, ^&&^p < 0.01 vs i3 within group (e). Data are mean + SEM.

In contrast to reward seeking behaviour, fear expression was more sensitive to adolescent conditioning over the course of DC training. In DC1 (Fig. 4a), ADSC-F rats displayed significantly more freezing than the other groups in response to the fear and the fear + safety cues (two-way RM ANOVA: cue: F(3, 180) = 153.4, p < 0.0001; group: F(3, 60) = 7.073, p = 0.0004; interaction: F(9, 180) = 5.84, p < 0.0001; post hoc comparisons for the fear cue: ADSC-F vs cxt-ctr and ADSC-R: p < 0.0001, ADSC-F vs ADSC-U: p = 0.0053; post hoc comparisons for the fear + safety cue: ADSC-F vs cxt-ctr and ADSC-R: p < 0.0001, ADSC-F vs ADSC-U: p = 0.0056). In addition, the ADSC-U group displayed more freezing in the fear + safety condition compared to the ADSC-R group (p = 0.0425).

As early as DC2 (Fig. 4b) the ADSC-R group already showed evidence of safety conditioning, as indicated by a significant suppression of freezing during the compound fear + safety cue compared to the fear cue alone (two-way RM ANOVA: cue: F(3, 180) = 601.2, p < 0.0001; group: F(3, 60) = 1.417, p = 0.2466; interaction: F(9, 180) = 2.265, p = 0.0200; post hoc comparison for fear vs fear + safety cues in the ADSC-R group: p = 0.0068). Furthermore, the ADSC-F group still froze significantly more to the fear and fear + safety cues compared to the cxt-ctr group (ADSC-F versus cxt-ctr: fear cue: p = 0.0413, fear + safety cue: p = 0.0161).

At DC3 (Fig. 4c), the cxt-ctr, ADSC-R and ADSC-U groups showed significantly less % time freezing during the fear + safety cue compared to the fear cue alone (two-way RM ANOVA: cue: F(3, 177) = 620.8, p < 0.0001; group: F(3, 59) = 1.228, p = 0.3075; interaction: F(9, 177) = 1.213, p = 0.2895; post hoc comparisons for fear vs fear + safety: cxt-ctr: p = 0.0324; ADSC-R: p = 0.0424; ADSC-U: p = 0.0060). This indicates significant safety expression in all groups except ADSC-F during DC3.

It was not until DC4 (Fig. 4d) that the ADSC-F group significantly reduced its freezing to the fear + safety cue, similar to the cxt-ctr and the ADSC-R groups (two-way RM ANOVA: cue: F(3, 177) = 936.9, p < 0.0001; group: F(3, 59) = 1.385, p = 0.2563; interaction: F(9, 177) = 1.644, p = 0.1059; post hoc comparisons for fear vs fear + safety: ADSC-F: p = 0.0004; cxt-ctr: p = 0.0159; ADSC-R: p = 0.0335). In the ADSC-U group, safety expression was no longer significant (p = 0.0620).

Fear extinction training significantly reduced freezing in all groups (Fig. 4e), but at different rates (two-way RM ANOVA: interval: F(3, 180) = 36.78, p < 0.0001; group: F(3, 60) = 0.4614, p = 0.7103; interaction: F(9, 180) = 2.417, p = 0.0130). The ADSC-F group showed a significant drop in freezing from i1 to i2, which was not observed until i3 in all other groups. Post hoc tests in control animals revealed a significant difference between i1 and the last two intervals (i3: p = 0.0023; i4: p = 0.0082), similar to the ADSC-R group (i1 vs i3: p = 0.0347; i1 vs i4: p < 0.0001). ADSC-U rats showed a significant reduction in freezing from i1 to i3 (p = 0.0012) and i4 (p < 0.0001). The ADSC-F group presented the steepest extinction curve, with i1 differing from i2, i3 and i4 (p < 0.0001 each). In addition, in the ADSC-R group, i2 and i3 also differed from i4 (p = 0.0003 and p = 0.0021, respectively), and i2 differed from i4 in ADSC-U rats (p = 0.0167).

Significant fear suppression to the fear + safety cue compared to the fear cue alone was still present in the extinction recall session in all groups, except for the ADSC-U group (Fig. 4f; two-way RM ANOVA: cue: F(3, 180) = 242.4, p < 0.0001; group: F(3, 60) = 0.7925, p = 0.5029; interaction: F(9, 180) = 0.81, p = 0.6076; post hoc comparisons for fear vs fear + safety: cxt-ctr: p = 0.0310, ADSC-R: p = 0.0267, ADSC-F: p = 0.0098). For all groups, freezing to the fear cue during extinction recall (Fig. 4f) was significantly lower than at the beginning of extinction training (i1; Fig. 4e), thus proving successful extinction (two-way RM ANOVA with i1, and fear cue in the extinction recall as the repeated factor: extinction stage: F(1, 60) = 119.3, p < 0.0001; group: F(3, 60) = 1.056, p = 0.3747; interaction: F(3, 60) = 0.6448, p = 0.5893).

Consistent throughout all DC and extinction recall sessions, each group froze significantly more during the fear and the fear + safety cue than to the reward cue or the safety cue alone (post hoc comparisons for DC1-4: p < 0.0001 within each group, not indicated in Fig. 4a–d,f), indicating very low levels of freezing in response to the reward or safety cue.

### Adolescent cued fear conditioning facilitated re-learning of the fear cue-shock association, but did not influence freezing to the first fear cue presentation

**Figure 5:**
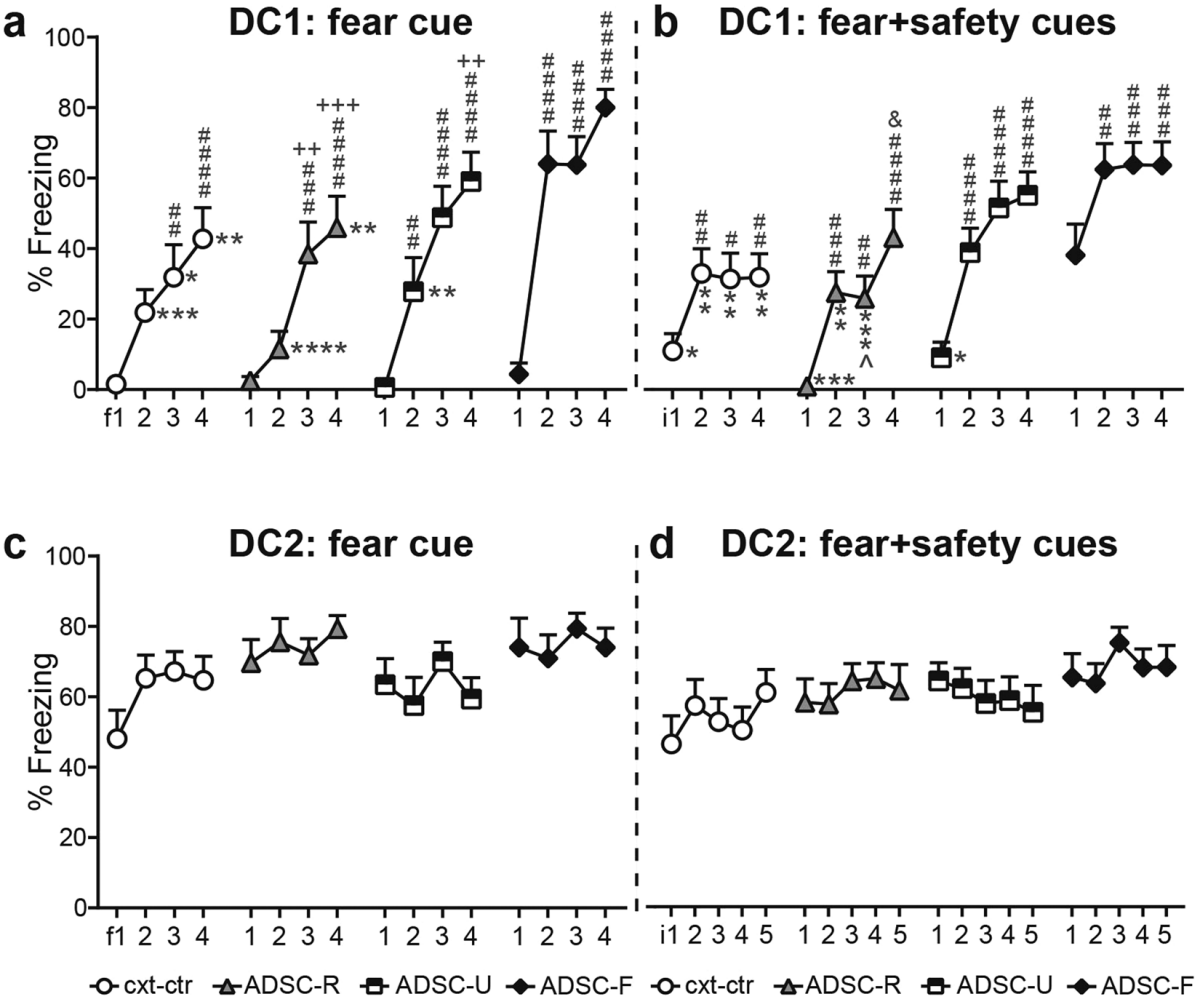
Adolescent fear conditioning facilitated re-learning of the fear cue-shock association, but did not influence freezing to the first fear cue presentation. Averaged % time freezing was analysed individually for each fear cue in DC1 (**a**) and DC2 (**c**). Freezing levels to the first fear cue, i.e. before any shock was presented are low in all groups, but noticeably accelerated after the first fear cue-shock pairing in the ADSC-F group (**a**). Fear + safety cues between two fear cue-shock presentations were averaged to form intervals (i) for DC1 (**b**) and DC2 (**d**). In DC1, ADSC-F rats showed high freezing levels in i1, after the first fear cue-shock presentation (**b**). At DC2, freezing levels did not change over the course of the session (**c**,**d**). Note that in DC2 fear + safety cues were presented before the first fear cue shock pairing, resulting in five intervals. f: fear cue; ****p < 0.0001, ***p < 0.001, **p < 0.01, *p < 0.05 between group difference vs ADSC-F within the same interval; ^p < 0.05 between group difference vs ADSC-U within the same interval; ^####^p < 0.0001, ^###^p < 0.001, ^##^p < 0.01, ^#^p < 0.05 within group difference vs f1 (**a**) or i1 (**b**); ^+++^p < 0.001, ^++^p < 0.01 within group difference vs f2 (a) or i2 (b), and ^&^p < 0.05 within group difference vs i3 (**b**). Data are mean + SEM.

To assess whether the high freezing levels in the ADSC-F group in DC1 was attributable to a memory from the adolescent learning experience or to facilitated re-learning, we analysed the development of the fear response over the course of the 4 fear cue trials in DC1 (Fig. 5a). Two-way RM ANOVA of % time freezing revealed a significant main effect for fear cues (F(3, 180) = 64.16, p < 0.0001), a significant main effect for groups (F3, 60) = 7.069, p = 0.0004) and a significant interaction (F(9, 180) = 2.693, p = 0.0058). Post hoc comparisons revealed differences in learning curves between the groups, despite a comparable starting point. Cxt-ctr rats increased their freezing levels from f1 to f3 (i.e. fear trials 1 to 3) (p = 0.0021) and f1 to f4 (p < 0.0001). The ADSC-R group increased its fear response from the first two fear cues to the last two fear cues (f1 vs f3: p = 0.0002; f1 vs f4: p < 0.0001; f2 vs f3: p = 0.0083; f2 vs f4: p = 0.0003). ADSC-U rats showed increases in freezing from f1 to f2 (p = 0.0073), f3 (p < 0.0001) and f4 (p < 0.0001) as well as from f2 to f4 (p = 0.0014). In contrast, adolescent fear conditioned rats (ADSC-F) reached a plateau starting at the second fear cue presentation (f1 vs f2, f3, f4: p < 0.0001 each).

Between-group differences of ADSC-F rats with all other groups were not observed at f1 (Fig. 5a), and only appeared from f2 onwards (f2: ADSC-F vs cxt-ctr: p = 0.0003, vs ASDC-R: p < 0.0001 and vs ADSC-U: p = 0.0024; f3: ADSC-F vs cxt-ctr: p = 0.0103; f4: ADSC-F vs cxt-ctr: p = 0.0018, vs ADSC-R: p = 0.0051).

Two-way RM ANOVA for each fear + safety cue interval revealed a significant main effect for interval (F(3, 180) = 41.03, p < 0.0001), a significant group effect (F(3, 60) = 8.157, p = 0.0001), but no significant interaction (F(9, 180) = 1.729, p = 0.0852). Post hoc comparisons revealed slight differences in freezing increases (Fig. 5b; cxt-ctr: i1 vs i2: p = 0.0058, i1 vs i3: p = 0.0123, i1 vs i4: p = 0.0099; ADSC-R: i1 vs i2: p = 0.0005, i1 vs i3: p = 0.0013, i1 vs i4: p < 0.0001, i3 vs i4: p = 0.0482; ADSC-U: i1 vs i2-i4: p < 0.0001 each; ADSC-F: i1 vs i2: p = 0.0018, i1 vs i3: p = 0.0008, i1 vs i4: p = 0.0009). The ADSC-F group differed from all other groups in i1 (cxt-ctr: p = 0.0205; ADSC-R: p = 0.0005; ADSC-U: p = 0.0112) and from the cxt-ctr group and the ADSC-R group in i2 (cxt-ctr: p = 0.0098; ADSC-R: p = 0.0013) and in i3 (cxt-ctr: p = 0.0034; ADSC-R: p = 0.0004). We also observed differences between the ADSC-R and ADSC-U groups in i3 (p = 0.0322) and between cxt-ctr and ADSC-F rats in i4 (p = 0.0044). Together, all groups showed the most pronounced increase in freezing from i1 to i2, with ADSC-F rats starting from a higher level in i1, after the first fear cue-shock pairing was presented.

Such dynamic alterations in freezing within a session was specific to DC1 and did not occur in DC2 (Fig. 5c,d; two-way RM ANOVA for fear cue: fear cue: F(3, 180) = 1.857, p = 0.1385; group: F(3, 60) = 2.37, p = 0.0794; interaction: F(9, 180) = 1.157, p = 0.3251; two-way RM ANOVA for fear + safety cue intervals: interval: F(4, 240) = 0.4575, p = 0.7669; group: F(3, 60) = 1.477, p = 0.2300; interaction: F(12, 240) = 1.146, p = 0.3236).

### Adolescent conditioning had minimal effects on contextual freezing

**Figure 6:**
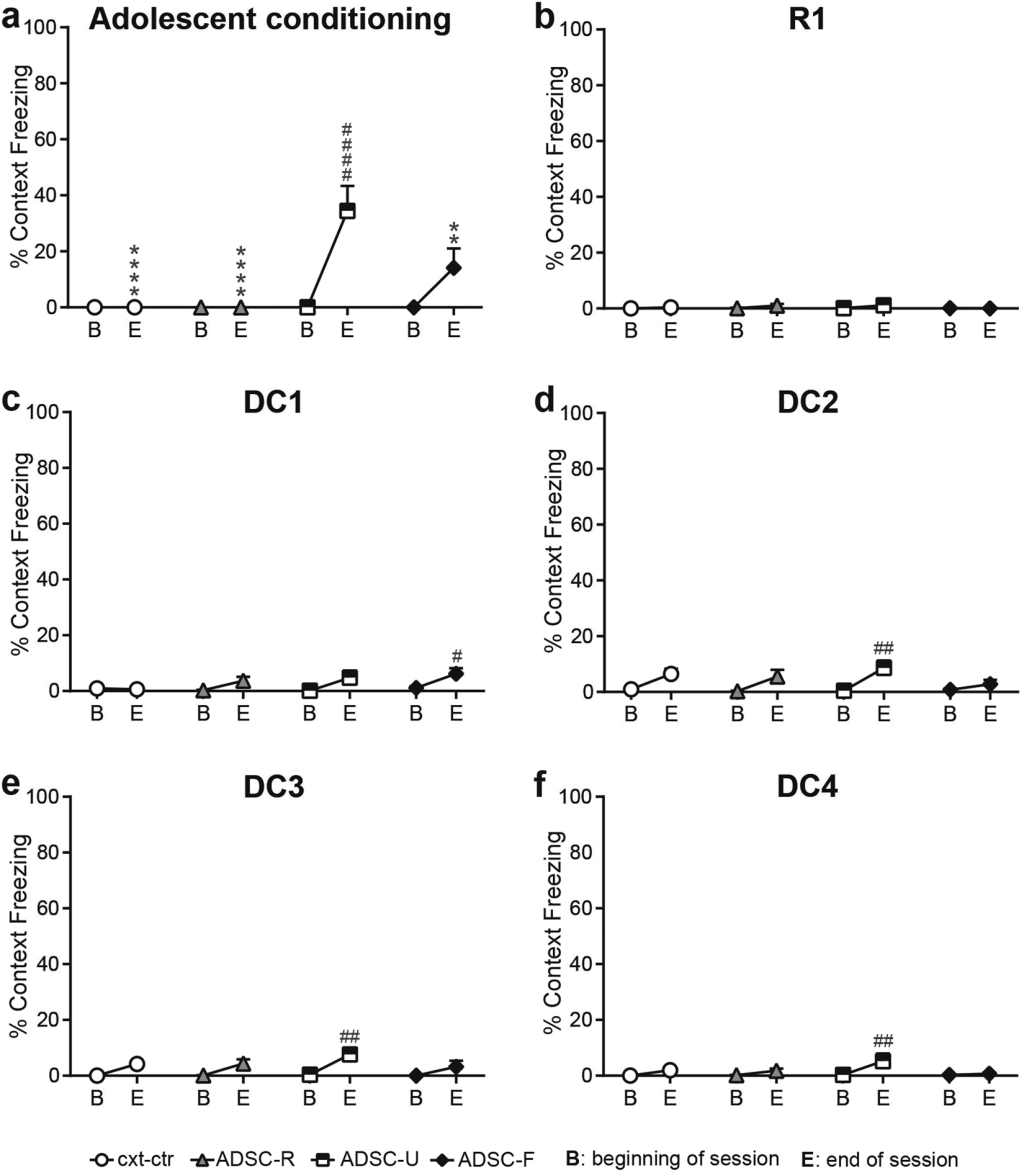
Adolescent conditioning had minimal effects on contextual freezing. Averaged % time freezing during the first (B) and last (E) two minutes of adolescent conditioning (**a**), R1 (**b**) and DC1-4 (**c**–**f**) was used as a measure for contextual fear. Immediately after adolescent conditioning, freezing was elevated in the ADSC-U group compared to its level in the beginning and compared to the other 3 groups (**a**). Freezing upon the first adult re-exposure to the chamber was absent in each group (**b**). Throughout discriminative conditioning, contextual freezing remained low but reached significance in the ADSC-F group in DC1 (**c**) and in the ADSC-U group in DC2-4 (**d**–**f**), when compared to the beginning of the session. ****p < 0.0001, **p < 0.01 between group difference vs ADSC-F within the same interval ^####^p < 0.0001, ^##^p < 0.01, ^#^p < 0.05 within group difference vs B. Data are mean + SEM.

To assess any possible freezing to the training context, we analyzed freezing levels during the first and last two minutes of a given session; these are segments in which no cues are presented. In the adolescent pre-conditioning session freezing was not observed in any group at the beginning of the session, but was significantly increased in the ADSC-U group at the end (Fig. 6a; segment of session: F(1, 60) = 18.47, p < 0.0001; group: F(3, 60) = 8.284, p = 0.0001; interaction: F(3, 60) = 8.284, p = 0.0001 post hoc comparisons: ADSC-S: B vs E: p < 0.0001, ADSC-S vs ctr-ctr and ADSC-R: p < 0.0001, ADSC-S vs ADSC-F: p = 0.0026). To confirm the ADSC-F group developed a freezing response to the fear cue during adolescence, percent time freezing was analyzed across the 4 fear cue-shock trials and was shown to significantly increase across trials (Supplementary Fig. S2).

Rats from neither shock group presented remarkable freezing in R1, the first adult re-exposure to the conditioning chamber. Despite a significant main effect for segment of session, freezing levels were low and post hoc tests failed to reach significance (Fig. 6b; segment of session: F(1, 60) = 5.8, p = 0.0191; group: F(3, 60) = 1.82, p = 0.1531; interaction: F(3, 60) = 1.214, p = 0.3123). Higher freezing was observed at the end of the session (E, last 2 mins) compared to the beginning (B, first 2 minutes) in DC1 for the ADSC-F group and in DC2-4 for the ADSC-U group (Fig. 6c–f; DC1: segment of session: F(1, 60) = 11.63, p = 0.0012; group: F(3, 60) = 1.791, p = 0.1585, interaction: F(3, 60) = 1.702, p = 0.1762, post hoc test: ADSC-F: B vs E: p = 0.365; DC2: segment of session: F(1, 60) = 22.7, p < 0.0001; group: F(3, 60) = 1.081, p = 0.3640; interaction: F(3, 60) = 1.335, p = 0.2714, post hoc test: ADSC-U: B vs E: p = 0.0018; DC3: segment of session: F(1, 59) = 23.37, p < 0.0001; group: F(3, 59) = 1.179, p = 0.3256; interaction: F(3, 59) = 0.8495, p = 0.4724, post hoc comparison: ADSC-U: B vs E: p = 0.0015; DC4: segment of session: F(1, 59) = 9.078, p = 0.0038; group: F(3, 59) = 1.583, p = 0.2030, interaction: F(3, 59) = 1.679, p = 0.1812, post hoc comparison: ADSC-U: B vs E: p = 0.0058). Observed differences remained within a small range and never persisted into the next session.

## Discussion

How previous experiences influence later learning performance is complex and events that we remember are rarely limited to one single association between a cue and an outcome. Moreover, unlike most experimental designs in rodents, real life memories are usually not formed on a clean slate, but rather interact with previous experiences^29^. In the present study, we investigated the influence of adolescent experiences on later learning of a discriminative conditioning paradigm well-validated by our laboratory^7^, which assesses the ability to discriminate among fear, safety and reward cues. In contrast to other studies which use adolescent interventions that are unrelated to the later behavioral read-out^16,18,20^, we pre-conditioned one component, either fear or reward, of the later more complex learning task. Our study revealed two main findings: (1) Adolescent conditioning determined the rate at which later safety expression was achieved, in which prior reward conditioning accelerated safety expression and prior fear conditioning delayed it. After adolescent footshock presentations unpaired to the light cue, we observed adult safety expression at an intermediate time point that faded by the end of DC training. (2) Adolescent fear conditioning accelerated adult fear expression, reward learning and fear extinction (summarized in Table 1).

**Table 1:** Summary of observed effects.

		Adolescence		
		Reward	Fear	Safety
Adulthood	Reward conditioning			
	Reward extinction			
	Fear conditioning			
	Fear extinction			
	Safety conditioning			

Arrows indicate differences from the cxt-ctr group, with angled arrows signifying a less prominent increase than straight arrows.

During adolescent conditioning, freezing increased in both the ADSC-F and ADSC-U groups (Figs 6a and S2). This effect did not persist into adulthood since freezing levels in r1, as well as the habituation session, and to the first fear cue presentation in DC1, i.e. before a footshock was applied in adulthood, were low in all groups. For the ADSC-F group, this indicates that the accelerated expression of freezing seen in DC1 is not caused by an increase in baseline fear or a strong memory of the adolescent experience. This is perhaps due to a weak memory of the adolescent experience, or the adolescent pre-conditioning may have primed the memory system to facilitate re-learning of the fear cue-shock association. Such so called ‘savings’ are mediated by the basal amygdala^30^. We further found that pre-conditioning did not support memory specificity, since freezing levels were also increased to the compound fear + safety cue in DC1 and significant fear suppression did not occur before DC4. Thus, the adolescent fear experience may have caused an increase in cue-dependent fear generalization presented in adulthood. We did observe evidence of fear generalization to the context in ADSC-F rats during DC1. However, the range of this effect was so small (below 10%) that it likely did not confound the cue responses. Interestingly, the generalization to the fear + safety cue did not diminish the ability to extinguish the fear cue; instead, adolescent paired fear conditioning accelerated fear extinction. These seemingly opposing results may be due to the DC sessions containing fear trials resulting in footshock intermixed with fear + safety trials resulting in no footshock, whereas extinction presented only the fear and reward cues with neither being reinforced. It is thus possible that adolescent fear conditioned rats have difficulty discerning the difference between a fear and fear + safety trial during DC, but during extinction do not have the same difficulty in recognizing that the fear cue is no longer paired with footshock. Since only the shock pre-exposed adolescent groups showed inconsistent safety expression, this possible lack in fear versus safety cue discrimination cannot be due to the effectiveness of the light cue as a safety cue, as both the context control and reward pre-conditioned groups demonstrated significant fear suppression in the presence of the light safety cue.

The lack of adult fear memory expression at the beginning of training in the adolescent fear conditioned group was surprising given the increased impact of adolescent, relative to adult, experiences^31,32^, and the longevity of fear memories^33^. However, the plasticity of a fear memory differs between adolescence and adulthood, in that a fear memory formed in adolescence can be erased by extinction^34^, whereas a fear memory formed in adulthood instead competes with a new memory for fear extinction^35^. Despite the well-described phenomenon of infantile amnesia, in which memories formed in the pre-weaning period rapidly fade (e.g.^36^), forgetting of adolescent Pavlovian memories^37^ and adult operant memories^38^ have been observed using even stronger conditioning protocols than ours. Chan *et al*.^36^ showed that even though infantile contextual fear memories appeared forgotten in adulthood, relearning was faster and did not require NMDA-receptor activation, unlike newly formed memories. Investigating the role of amygdalar NMDA-receptors in relearning an association initially acquired in adolescence, would therefore be interesting for future studies.

On a molecular level, a potential mediator would have to be sensitive to adolescent experiences and/or present a developmental peak at the time when our adolescent conditioning took place, plus be involved in emotional learning of different valences. Among these candidates is BDNF, which promotes maturation of the adolescent visual cortex^39^. In the amygdala and the hippocampus, adolescent stress experiences increase BDNF levels^40^. Further, and in addition to its role in appetitive^41^ and aversive conditioning^42^, BDNF levels in the hippocampus increase following safety learning^25^. Likewise, the dopaminergic system presents similar features. Dopamine receptor D1 (DRD1) density in the prefrontal cortex peaks in adolescence^43^, and we have previously shown that adult safety learning requires activity in the infralimbic subregion of the prefrontal cortex^44^ and an optimal level of D1 receptor activation in the basal amygdala^45^.

Alternatively, the cannabinoid system may also be contributing to the observed effects, since it presents with developmental peaks at the time when our adolescent conditioning takes place^46^. Cannabinoid receptor 1 (CB1R) expression in the PFC is highest at P29 in rats^47^, which corresponds to our adolescent conditioning time window of P30-32. In adolescent rats, the BLA cannabinoid system mediates social play behaviour^48^, demonstrating its involvement in the development of reward-associated behaviors. And antagonising the CB1 receptor in adulthood, either systemically or locally in the dorsal hippocampus, impairs safety learning in a step-down avoidance task^49^, and systemic blockade of CB1R facilitates extinction of cocaine seeking^50^. Finally, CB1R-knock out mice show increased stress vulnerability^51^. Moreover, knock out of CB1R, specifically on dopamine receptor 1 expressing neurons, diminishes memory specificity^52^, and CB1R-knock out mice present with reduced hippocampal BDNF^53^. Together, these results in reward, fear and safety paradigms point to BDNF, the dopaminergic and cannabinoid systems, and/or interactions between them as potential mediators of our results. Given the complexity of the DC-task and the multitude of processes taking place during the transition from adolescence to adulthood, an interaction of different systems is likely.

Apart from molecular changes possibly involved, alterations at the electrophysiological level may be contributing as well. Sangha *et al*.^7^ previously showed a population of neurons in the basal amygdala responding to both the safety and reward cues with the same level of excitation or inhibition, demonstrating an overlap between safety and reward circuits in the amygdala^7^. Other regions processing reward cues, like the VTA, paraventricular thalamus and nucleus accumbens, also respond to fear cues^6,11,12^. This provides a potential anatomical basis for the observed safety learning facilitation in the reward-pre-conditioned group and the facilitated reward learning in cued fear-pre-conditioned rats. Additionally, during development, there are changes in the intrinsic excitability of the amygdala^54,55^ and these changes determine the timing of critical periods^56^. Stress experienced during this period alters basal amygdala excitability^57^, and adolescent stress combined with adult stress leads to long term changes in hippocampal excitability observed as long as one month later, whereas changes induced by only one stressor are transient^58^. It is therefore possible that adolescent pre-conditioning altered amygdalar excitability, thereby facilitating later safety learning in the ADSC-R group, and fear and reward learning in the ADSC-F group. In order to narrow down the specificity of our results to adolescent pre-conditioning, it would be interesting to investigate whether similar effects would be observed when pre-conditioning instead took place in adulthood, where the molecular makeup is different.

Since we used aversive footshocks as the unconditioned stimulus, adolescent conditioning might not only induce learning processes, but also a stress response. A hallmark of stress-induced memory disturbances characteristic of PTSD models is fear generalization to the non-threatening chamber in cued fear conditioning paradigms (e.g.^20^). Although we observed contextual generalization in the ADSC-F and ADSC-U groups at the end of the DC sessions, its extent was low, especially in relation to the degree of freezing seen in response to the fear or the fear + safety cue. Moreover, this effect never persisted into the next session, so we do not consider our adolescent conditioning as traumatic. Unspecific stress effects contributing to our results are therefore unlikely. Depending on its particular characteristics, a stressor can be beneficial and support later memory formation^59^, even when experienced in adolescence^60^. However, the faster rates of fear and reward learning and fear extinction observed in the adolescent fear conditioned group cannot be attributed to stress effects caused by the footshocks. ADSC-U rats, who experienced the same number of footshocks at the same intensity did not express the same increased rates of learning. Both groups that received footshocks during adolescence (ADSC-F, ADSC-U), however, showed impaired safety conditioning in adulthood. Adolescent fear conditioned rats did not show fear suppression to the compound fear + safety cue compared to the fear cue alone until the last DC session. Since the regions mediating extinction (basolateral amygdala^61,62^ infralimbic cortex^62^) and safety (basolateral amygdala^7^, infralimbic cortex^44^) overlap at least partially, one could expect that a steep extinction curve would co-occur with improved safety learning. However, we have shown previously the presence of fear-specific and safety-specific neuronal populations in the BLA^7^. And even though many of these safety-specific neurons do become responsive to the fear cue during fear extinction, they do not preferentially become fear cue responsive during extinction, when the fear cue no longer signals the occurrence of a footshock^8^. This suggests some divergence in the mechanisms mediating fear extinction and safety conditioning in our paradigm, possibly explaining the dissociation between fear extinction and safety learning in adolescent fear conditioned rats.

Rats in the ADSC-U group that experienced unpaired footshocks in adolescence showed lower freezing during the fear + safety cue during DC3, but not during DC4. Loss of memory specificity is frequently seen as a consequence of strong training protocols^63–65^ or after traumatic, adolescent stress experiences^20^. Our shock protocol with four presentations of 0.45 mA could be considered as mild, compared to the studies cited above, which used seven presentations of 0.9 mA^64^. Interestingly, mild stress like this was enough to impact safety learning; whether the shock was paired or unpaired with a cue, rats showed a lack of fear suppression to the compound fear + safety cue, although with different temporal dynamics. The ADSC-U group showed inconsistent expression of safety across the sessions, whereas the ADSC-F group clearly exhibited delayed development of safety expression. This illustrates how sensitive safety conditioning is to environmental stimulation. In PTSD patients the ability to deploy and process safety cues is reduced^66,67^, and another recent study in humans showed that diminished safety learning after trauma serves as an early predictor for PTSD symptom development^68^. It will therefore be interesting for future studies to investigate how conditioned safety and other parameters of our paradigm interact when a severe stressor is presented.

Apart from safety learning, fear extinction is also impaired in patients with PTSD^69–71^, and since the experience of a trauma is a precondition for the diagnosis, most animal models of this disease use severe priming stressors that later lead to pronounced behavioural disturbances^18,31^. Here, we were able to model diminished safety learning, without impairing extinction using mild fear conditioning, which now enables us to better dissect the underlying maladaptive memory processes in this disorder. Developing animal models using a range in stressor severity across different developmental stages is essential in understanding the nuances of how prior experiences can influence the ability to regulate fear and motivated behaviors in response to cues representing reward, fear, explicit safety or fear extinction. This will ultimately aid the development of patient tailored therapies for heterogeneous and complex disorders, like PTSD.

## Materials and Methods

### General

64 male Long Evans rats were purchased from Envigo (Indianapolis, IN, USA) and arrived at Purdue University’s Department of Psychological Science’s animal facility at postnatal day (P) 21 and maintained on a 12 h light/dark cycle with lights on at 9am. Experiments were carried out during the light phase. Upon arrival at our facility P21 rats were pair-housed and remained pair-housed during adolescent pre-conditioning and until adulthood with *ad libitum* access to water and food. During this time rats were paired according to the same conditioning group (e.g. 2 rats assigned to cxt-ctr were housed together). Five days before adult DC training (r1), animals were re-housed into single housing. This was done to be consistent with other experiments in our laboratory in which adult rats are typically single-housed upon arrival before they undergo DC training. After the first day of reward conditioning (r1), rats were maintained on 22 g of food pellets per day, which were delivered to their home cages after each training session. This amount does not result in loss of body weight. Rats were handled for 3 days prior to adolescent pre-conditioning and for 5 days prior to adult conditioning. Body weight was monitored daily during experiments and weekly between adolescent and adult conditioning to assess potential unspecific stress effects induced by adolescent conditioning/footshock experience. All experiments were in accordance with and approved by the Purdue Animal Care and Use Committee.

#### Apparatus

Adolescent pre-conditioning and adult discriminative conditioning took place in the same Plexiglas chamber (32 cm × 25 cm × 30 cm). The chamber contained a grid floor for shock delivery, a port 2 cm above the floor in the center of one wall for sucrose delivery. Two lights flanking the port provided the light cue and a “tweeter” speaker (ENV-224BM) located on the same wall provided the auditory cues (Med Associates, ST Albans, VT). A house light located on the opposite wall provided constant light (28 V, 100 mA). The chamber was enclosed in a sound attenuating chamber and a camera mounted on the door of this chamber video recorded the sessions for later offline analyses.

### Behavioural training

#### Adolescent pre-conditioning

Rat pairs were randomly assigned to one of four groups (n = 16/group, Fig. 1):

- Context control (cxt-ctr): rats were exposed once to the conditioning chamber for 30 minutes at P30
- Adolescence-reward (ADSC-R): rats received 25 pairings of the reward cue (tone: 3 kHz, 20 s, continuous) and a 10% sucrose solution each day from P30 to P32 (i.e. total 3 sessions).
- Adolescence-unpaired safety (ADSC-U): rats received 4 footshocks (0.5 s, 0.45 mA) that were explicitly unpaired with 4 safety cue presentations (light: 28 V, 100 mA, 20 s, continuous) on P30.
- Adolescence-fear (ADSC-F): rats were presented with 4 fear cue-footshock pairings (tone: 11 kHz, 20 s, 200 ms on, 200 ms off; shock: 0.5 s, 0.45 mA) on P30.

For the ADSC-R and the ADSC-F groups, the reward and fear cues used in adolescent pre-conditioning (3kHZ and 11kHZ, respectively) were again used later in the DC-task as the reward and fear cues. In the ADSC-U group, the footshock was unpaired with the later safety cue (light) instead of the fear cue to avoid potential safety conditioning to the fear cue.

#### Discriminative conditioning (DC) in adulthood

Discriminative conditioning was performed as published previously^7,8,44,45,72^. In brief, beginning at P75, rats received 5 reward training sessions followed by 1 habituation, 4 discriminative conditioning, 1 extinction training and 1 extinction recall session on consecutive days with 1 session per day. During reward conditioning, rats received 25 pairings of the reward cue (tone: 3 kHz, 20 s, continuous) and a 10% sucrose solution delivered in the port. In the habituation session, reward conditioning continued with 25 trials, in addition rats were (re)familiarized with the later safety and fear cues by presenting 5 unreinforced fear cues (tone: 11 kHz, 20 s, 70 dB, 200 ms on, 200 ms off) and 5 safety cues (light: 28 V, 100 mA, continuous). Adding the safety cue to the house light leads to a 40% increase in light intensity (from 7 lux to 11 lux) within the chamber. During discriminative conditioning, rats were trained to 4 stimulus combinations. These were reward (15 trials of reward cue–liquid sucrose pairings), fear (4 trials of fear cue–footshock (0.5 s, 0.45 mA) pairings), fear + safety (15 trials of the compound fear + safety cue, unreinforced, to assess the ability of the safety cue to suppress fear), and safety (10 trials of the safety cue presented alone to assess if fear is present). Extinction training included 20 unreinforced presentations, each, of the fear and reward cues, and extinction recall contained 10 reward (no sucrose), 10 fear (no footshock), 5 compound fear + safety, and 5 safety cues. In each session trials were presented in a pseudorandomized order and at a variable inter-trial interval of 90–130 s (reward pre-conditioning and habituation), 100–140 s (DC), 60–120 s (extinction training and recall). Sessions were flanked with 2 (habituation), 5 (reward pre-conditioning, extinction training and extinction recall) or 10 (DC) minute epochs, in which no stimuli were presented.

### Data analyses

Two parameters were assessed during each cue presentation: (1) time spent in the port was detected automatically by infrared sensors located in the port and served as a measure for reward seeking, and (2) time spent freezing (immobility except for respiration) was assessed manually as a read out for fear. Behaviour was scored off-line by 3 different experimenters who were blind to group affiliation and had an inter-rater correlation of r > 0.8. Moreover, groups were balanced across experimenters. The video files of DC3 and DC4 of one rat from the ADSC-R group were corrupted and could therefore not be analysed. For the first reward conditioning session and extinction training, blocks of 5 fear or reward cues were averaged to form the intervals 1–4 (i1-4). For DC1 and DC2 the development of behavioural responses to the individual fear (f1-4), and fear + safety cues was assessed additionally, in a trial by trial manner. Since behavioural changes are expected to occur in response to the aversive footshocks, and the number of fear and fear + safety trials (4 vs 15) is very different, we averaged fear + safety cues between two fear cues to form intervals (i). In DC2, unlike DC1, fear + safety cues were presented before the first fear cue-shock pairing, resulting in five intervals. Additionally, fear in the first and last two minutes (segment of session) of adolescent pre-conditioning as well as in R1 and DC1-4 in adulthood were analyzed. Freezing and reward seeking, expressed as % time, were analysed with two-way repeated measures (RM) ANOVAs (group x cue or group x session or interval for the reward training or extinction training, and fear + safety cue intervals in DC1 and 2) using GraphPad Prism. Behavioural responses to the fear and safety cues during the habituation were assessed with a one-way ANOVA. If applicable, ANOVAs were followed by two-sided Tukey´s multiple comparison tests for post hoc analyses. The alpha level was set to 0.05. Data in diagrams are displayed as mean + standard error of mean (SEM). Additionally, effect sizes (Cohen’s d) for significant *post hoc* comparisons are provided in the Supplementary Table S1 to better interpret the magnitude of observed effects.

## Electronic supplementary material

Supplemental materials

Dataset 1

## Data Availability

The datasets generated during and/or analysed during the current study are available as supplemental materials.
