# Supplementary material for: Adolescent conditioning affects rate of adult fear, safety and reward learning during discriminative conditioning
Source: Sci Rep. 2018 Nov 23;8:17315. doi: 10.1038/s41598-018-35678-9 (PMC6251908; doi:10.1038/s41598-018-35678-9)
Supplement: Supplementary file 1 — Supplemental materials [file 41598_2018_35678_MOESM1_ESM.docx]

**Adolescent conditioning affects rate of adult fear, safety and reward learning during discriminative conditioning**

**Iris Müller *^a, b^, Alyson L Brinkman^a^, Elizabeth M Sowinski^a^, Susan Sangha*^a,b^**

SUPPLEMENTARY MATERIAL

Adolescent conditioning potentially impacts the response to the first adult re-exposure to the cues, and we observed differences in the rate of fear expression during DC1 in rats previously conditioned to this association (Figure 5a). We therefore analysed freezing levels to the first five presentations of the three cues in adulthood.

A two-way RM ANOVA for reward cues during the first reward session indicated a significant group effect (figure S1a; F(3, 60) = 2.794, p = 0.0479), but no cue effect (F(4, 240) = 1.729, p = 0.1443) and no interaction (F(12, 240) = 1.59, p = 0.0950). Post hoc comparisons reached significance only in the first trial (cxt-ctr vs. ADSC-F: p = 0.0028, ADSC-R vs ADSC-F: p = 0.0015, ADSC-U vs ADSC-F: p = 0.0121). From the second reward cue onwards, group differences were not observed anymore.

For the habituation session, a two-way RM ANOVA revealed a significant main effect for the fear cues (F(4, 240) = 12.14, p < 0.0001), but no group effect (F(3, 60) = 0.5771, p = 0.6323) or interaction (F(12, 240) = 1.096, p = 0.3644). Despite similar responses in all four groups, Tukey´s post hoc comparisons revealed significant differences within the ADSC-R and ADSC-F groups during the pseudo-randomized presentations of the fear trials (no shock) embedded within presentations of the safety cue and reward cues (ADSC-R: trial 1 vs 2: p = 0.0207; trial 1 vs 3: p = 0.0037; trial 1 vs 4: p = 0.0091; ADSC-F: trial 1 vs 2: p = 0.0439; trial 1 vs 3: p = 0.0068; trial 1 vs 4 and 5: p < 0.0001 each). No significant differences in freezing levels were observed to the safety trials (F(4, 240) = 0.5299, p = 0.7138; group: F(3, 60) = 0.4337, p = 0.7296; interaction: F(12, 240) = 1.284, p = 0.2282). By the fourth trial presentation, freezing levels in all groups had reached a minimum.

**Figure S1**. **Initial freezing to the first re-exposure of the cues**. ADSC-F rats froze slightly more to the very first reward cue presentation (S1a). In habituation, the ADSC-R and ADSC-F groups showed a reduction in freezing to the fear cue over the course of five presentations. No differences were observed when the safety cues were presented. **p < 0.01, *p < 0.05 vs ADSC-F; ^####^p < 0.0001, ^##^p < 0.01, ^#^p < 0.05 vs fear cue trial 1. Data are mean +SEM.

In addition to the assessment of baseline and conditioning induced freezing levels in adolescence (Figure 6a), we analysed fear development over the course of the conditioning session (Figure S2). Since the ADSC-U group experiences four light cues and then four footshocks, freezing was measured in the 20s interval before a shock was presented to mirror the measurements in the ADSC-F group. This was a stimulus-free period and therefore reflected contextual freezing. Adolescent reward conditioning did not induce freezing (ADSC-R). In the ADSC-U group, freezing increased continuously over the course of the session, similar to the response to the fear cue in the ADSC-F group (two-way RM ANOVA: trial: F(3, 135) = 23.91, p < 0.0001; group: F(2, 45) = 7.869, p = 0.0012; interaction: F(6, 135) = 6.452, p < 0.0001; Tukey’s post hoc comparisons: ADSC-U: trial 1 vs 3: p = 0.0153, trial 1 vs 4: p < 0.0001, trial 2 vs 4: p < 0.0001, trial 3 vs 4: p = 0.0305; ADSC-F: trial 1 vs 3: p = 0.0005, trial 1 vs 4: p < 0.0001, trial 2 vs 3: p = 0.0033, trial 2 vs 4: p < 0.0001, trial 3 vs 4: p = 0.0073; in addition trials 3 and 4 in the ADSC-R group differed from the ADSC-U and ADSCF group: p < 0.005, not indicated in the diagram).

**
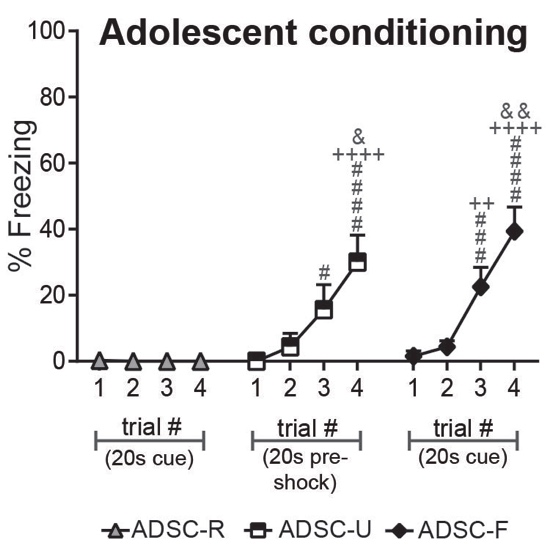
Figure S2. Fear development over the course of adolescent conditioning**. Reward conditioning did not induce freezing in the ADSC-R group. In the ADSC-U and ADSC-F groups, freezing increased with the number of shock and fear cue-shock presentations, respectively. Since, in the ADSC-U group cues and shocks were unpaired, we measured the 20s pre-shock interval to best match the assessment in the ADSC-F group. ^####^p < 0.0001, ^###^p < 0.001, ^#^p < 0.05 vs trial 1;^++++^p < 0.0001, ^++^p < 0.01 vs trial 2, ^&&^p < 0.01, ^&^p < 0.05 vs trial 3, all are within group differences. Data are mean+SEM.

**Supplementary Table 1: Effect sizes for rate of reward, fear extinction and safety expression.** Since the magnitude and relevance of statistically significant effects are difficult to assess from the p-values alone, this table depicts Cohen’s d as a measure for the effect size. Cohen (1988) defined the magnitude of effects as small (d = 0.2), medium (d = 0.5) and high (d = 0.8 and above). Of note, with the exception of r1 vs r4 during reward conditioning in the ADSC-R group, we did not observe small effects, supporting the validity of the observed findings.

| **session** | **cue/group** | **comparison** | **p-value** | **Cohen’s d** | **magnitude of effect** | **figure** |
| --- | --- | --- | --- | --- | --- | --- |
| reward  conditioning  (% Port) | ADSC-R | r1 vs r4 | 0.0481 | 0.477 | small | figure 2 |
|  | ADSC-R | r1 vs r5 | 0.0189 | 0.666 | medium |  |
|  | ADSC-U | r1 vs r5 | <0.0001 | 1.141 | high |  |
|  | ADSC-F | r1 vs r2 | 0.0145 | 0.843 | high |  |
|  | ADSC-F | r1 vs r3 | 0.0005 | 0.840 | high |  |
|  | ADSC-F | r1 vs r4 | 0.0065 | 0.686 | medium |  |
|  | ADSC-F | r1 vs r5 | <0.0001 | 1.014 | high |  |
| DC1  (% Freezing) | Fear | cxt-ctr vs ADSC-F | <0.0001 | 1.415 | high | figure 4 |
|  | Fear | ADSC-R vs ADSC-F | <0.0001 | 1.439 | high |  |
|  | Fear | ADSC-U vs ADSC-F | 0.0053 | 0.882 | high |  |
|  | Fear+Safety | cxt-ctr vs ADSC-F | <0.0001 | 1.187 | high |  |
|  | Fear+Safety | ADSC-R vs ADSC-F | <0.0001 | 1.429 | high |  |
|  | Fear+Safety | ADSC-U vs ADSC-F | 0.0056 | 0.756 | medium |  |
|  | Fear+Safety | ADSC-R vs ADSC-U | 0.0425 | 0.662 | medium |  |
| DC2  (% Freezing) | ADSC-R | Fear vs Fear+Safety | 0.0068 | 1.102 | high | figure 4 |
|  | Fear | cxt-ctr vs ADSC-F | 0.0413 | 0.622 | medium |  |
|  | Fear+Safety | cxt-ctr vs ADSC-F | 0.0161 | 0.713 | medium |  |
| DC3  (% Freezing) | cxt-ctr | Fear vs Fear+Safety | 0.0324 | 1.088 | high | figure 4 |
|  | ADSC-R | Fear vs Fear+Safety | 0.0424 | 0.667 | medium |  |
|  | ADSC-U | Fear vs Fear+Safety | 0.0060 | 1.282 | high |  |
| DC4  (% Freezing) | cxt-ctr | Fear vs Fear+Safety | 0.0159 | 1.004 | high | figure 4 |
|  | ADSC-R | Fear vs Fear+Safety | 0.0335 | 0.913 | high |  |
|  | ADSC-F | Fear vs Fear+Safety | 0.0004 | 1.094 | high |  |
| Extinction  training  (% Freezing) | cxt-ctr | i1 vs i3 | 0.0023 | 1.158 | high | figure 4 |
|  | cxt-ctr | i1 vs i4 | 0.0082 | 0.606 | medium |  |
|  | ADSC-R | i1 vs i3 | 0.0347 | 0.699 | medium |  |
|  | ADSC-R | i1 vs i4 | <0.0001 | 1.439 | high |  |
|  | ADSC-R | i2 vs i4 | 0.0003 | 1.144 | high |  |
|  | ADSC-R | i3 vs i4 | 0.0021 | 0.872 | high |  |
|  | ADSC-U | i1 vs i3 | 0.0012 | 0.855 | high |  |
|  | ADSC-U | i1 vs i4 | <0.0001 | 0.899 | high |  |
|  | ADSC-U | i2 vs i4 | 0.0167 | 0.603 | medium |  |
|  | ADSC-F | i1 vs i2 | <0.0001 | 1.078 | high |  |
|  | ADSC-F | i1 vs i3 | <0.0001 | 1.200 | high |  |
|  | ADSC-F | i1 vs i4 | <0.0001 | 1.269 | high |  |
| Extinction recall  (% Freezing) | cxt-ctr | Fear vs Fear+Safety | 0.0310 | 0.944 | high | figure 4 |
|  | ADSC-R | Fear vs Fear+Safety | 0.0267 | 0.873 | high |  |
|  | ADSC-F | Fear vs Fear+Safety | 0.0098 | 0.843 | high |  |
| DC1  fear cue  (% Freezing) | cxt-ctr | f1 vs f3 | 0.0021 | 0.806 | high | figure 5 |
|  | cxt-ctr | f1 vs f4 | <0.0001 | 1.151 | high |  |
|  | ADSC-R | f1 vs f3 | 0.0002 | 0.949 | high |  |
|  | ADSC-R | f1 vs f4 | <0.0001 | 1.145 | high |  |
|  | ADSC-R | f2 vs f3 | 0.0083 | 0.666 | medium |  |
|  | ADSC-R | f2 vs f4 | 0.0003 | 1.051 | high |  |
|  | ADSC-U | f1 vs f2 | 0.0073 | 0.696 | medium |  |
|  | ADSC-U | f1 vs f3 | <0.0001 | 1.330 | high |  |
|  | ADSC-U | f1 vs f4 | <0.0001 | 1.776 | high |  |
|  | ADSC-U | f2 vs f4 | 0.0014 | 0.912 | high |  |
|  | ADSC-F | f1 vs f2 | <0.0001 | 1.616 | high |  |
|  | ADSC-F | f1 vs f3 | <0.0001 | 1.903 | high |  |
|  | ADSC-F | f1 vs f4 | <0.0001 | 3.429 | high |  |
|  | f2 | cxt-ctr vs ADSC-F | 0.0003 | 1.317 | high |  |
|  | f2 | ADSC-R vs ADSC-F | <0.0001 | 1.762 | high |  |
|  | f2 | ADSC-U vs ADSC-F | 0.0024 | 0.957 | high |  |
|  | f3 | cxt-ctr vs ADSC-F | 0.0103 | 0.919 | high |  |
|  | f4 | cxt-ctr vs ADSC-F | 0.0018 | 1.279 | high |  |
|  | f4 | ADSC-R vs ADSC-F | 0.0051 | 1.161 | high |  |
| DC1  fear+safety cue  (% Freezing) | cxt-ctr | i1 vs i2 | 0.0058 | 0.957 | high | figure 5 |
|  | cxt-ctr | i1 vs i3 | 0.0123 | 0.791 | medium |  |
|  | cxt-ctr | i1 vs i4 | 0.0099 | 0.888 | high |  |
|  | ADSC-R | i1 vs i2 | 0.0005 | 1.059 | high |  |
|  | ADSC-R | i1 vs i3 | 0.0013 | 0.933 | high |  |
|  | ADSC-R | i1 vs i4 | <0.0001 | 1.258 | high |  |
|  | ADSC-R | i3 vs i4 | 0.0482 | 0.669 | medium |  |
|  | ADSC-U | i1 vs i2 | <0.0001 | 0.855 | high |  |
|  | ADSC-U | i1 vs i3 | <0.0001 | 1.073 | high |  |
|  | ADSC-U | i1 vs i4 | <0.0001 | 1.203 | high |  |
|  | ADSC-F | i1 vs i2 | 0.0018 | 0.956 | high |  |
|  | ADSC-F | i1 vs i3 | 0.0008 | 0.876 | high |  |
|  | ADSC-F | i1 vs i4 | 0.0009 | 0.814 | high |  |
|  | i1 | cxt-ctr vs ADSC-F | 0.0205 | 0.947 | high |  |
|  | i1 | ADSC-R vs ADSC-F | 0.0005 | 1.474 | high |  |
|  | i1 | ADSC-U vs ADSC-F | 0.0112 | 1.039 | high |  |
|  | i2 | cxt-ctr vs ADSC-F | 0.0098 | 1.021 | high |  |
|  | i2 | ADSC-R vs ADSC-F | 0.0013 | 1.297 | high |  |
|  | i3 | cxt-ctr vs ADSC-F | 0.0034 | 1.187 | high |  |
|  | i3 | ADSC-R vs ADSC-F | 0.0004 | 1.498 | high |  |
|  | i3 | ADSC-R vs ADSC-U | 0.0322 | 0.922 | high |  |
|  | i4 | cxt-ctr vs ADSC-F | 0.0044 | 1.187 | high |  |

[Cohen, Jacob](https://en.wikipedia.org/wiki/Jacob_Cohen_(statistician)) (1988). [*Statistical Power Analysis for the Behavioral Sciences*](https://books.google.com/books?id=2v9zDAsLvA0C&pg=PP1). Routledge. [ISBN](https://en.wikipedia.org/wiki/International_Standard_Book_Number) [1-134-74270-3](https://en.wikipedia.org/wiki/Special:BookSources/1-134-74270-3)
